# Roles of non-coding RNA in diabetic cardiomyopathy

**DOI:** 10.1186/s12933-024-02252-9

**Published:** 2024-06-29

**Authors:** Xi Yao, Xinyue Huang, Jianghua Chen, Weiqiang Lin, Jingyan Tian

**Affiliations:** 1 Kidney Disease Center, The First Affiliated Hospital, Zhejiang University School of Medicine, Hangzhou, 310003 China; 2 International School of Medicine, International Institutes of Medicine, The 4th Affiliated Hospital of Zhejiang University School of Medicine, Yiwu, 322000 China; 3 Department of Endocrine and Metabolic Diseases, Shanghai Institute of Endocrine and Metabolic Diseases, Ruijin Hospital, Shanghai Jiao Tong University School of Medicine, Shanghai, 200025 China; 4 Shanghai National Clinical Research Center for Metabolic Diseases, Key Laboratory for Endocrine and Metabolic Diseases of the National Health Commission of the PR China, Shanghai Key Laboratory for Endocrine Tumor, Clinical Trials Center, Ruijin Hospital, Shanghai Jiao Tong University School of Medicine, Shanghai, 200025 China

**Keywords:** Non-coding RNA, Diabetic cardiomyopathy, Pathogenesis

## Abstract

In recent years, the incidence of diabetes has been increasing rapidly, posing a serious threat to human health. Diabetic cardiomyopathy (DCM) is characterized by cardiomyocyte hypertrophy, myocardial fibrosis, apoptosis, ventricular remodeling, and cardiac dysfunction in individuals with diabetes, ultimately leading to heart failure and mortality. However, the underlying mechanisms contributing to DCM remain incompletely understood. With advancements in molecular biology technology, accumulating evidence has shown that numerous non-coding RNAs (ncRNAs) crucial roles in the development and progression of DCM. This review aims to summarize recent studies on the involvement of three types of ncRNAs (micro RNA, long ncRNA and circular RNA) in the pathophysiology of DCM, with the goal of providing innovative strategies for the prevention and treatment of DCM.

## Introduction

Diabetic cardiomyopathy (DCM) is a type of diabetic heart disease with abnormal myocardial structure and function in diabetic patients without other cardiovascular diseases (such as coronary heart disease, hypertension, severe valvular disease, and congenital heart disease) [1, 2]. Individuals with type 2 diabetes mellitus (T2DM) are estimated to face a 75% higher risk of cardiovascular mortality or hospitalization for heart failure compared with patients without diabetes [3]. The underlying mechanisms of DCM, which encompass altered metabolism, mitochondrial dysfunction, oxidative stress, inflammation, cardiac fibrosis, cell death, and extracellular matrix remodeling, have not been fully elucidated and remain subject to debate [4]. Disruption in energy substrate utilization [5–7], calcium and sodium homeostasis disorder [8–10], insulin resistance [11, 12], potential involvement of epicardial fat [13–16], and endothelial dysfunction [17–19] are believed to contribute to the onset and progression of DCM.

Non-coding RNAs (ncRNAs) are highly functional and dynamic nucleic acids that do not encode proteins. They include RNAs with specific functions, such as rRNAs, tRNAs, snRNAs, snoRNAs, and microRNAs (miRNAs), as well as RNAs with unknown functions. Long non-coding RNAs (lncRNAs) and circular RNAs (circRNAs) are the novel members of non-coding RNA family, but their functions and regulatory mechanisms are still not fully understood. Accumulating evidence indicates that ncRNAs play a crucial role in the regulation of endothelial cells, vascular and smooth muscle cells, cardiac metabolism, ischemia and inflammation. This indicates that ncRNAs hold significant potential in the diagnosis, evaluation, and treatment of DCM.

## MicroRNAs

**Fig. 1:**
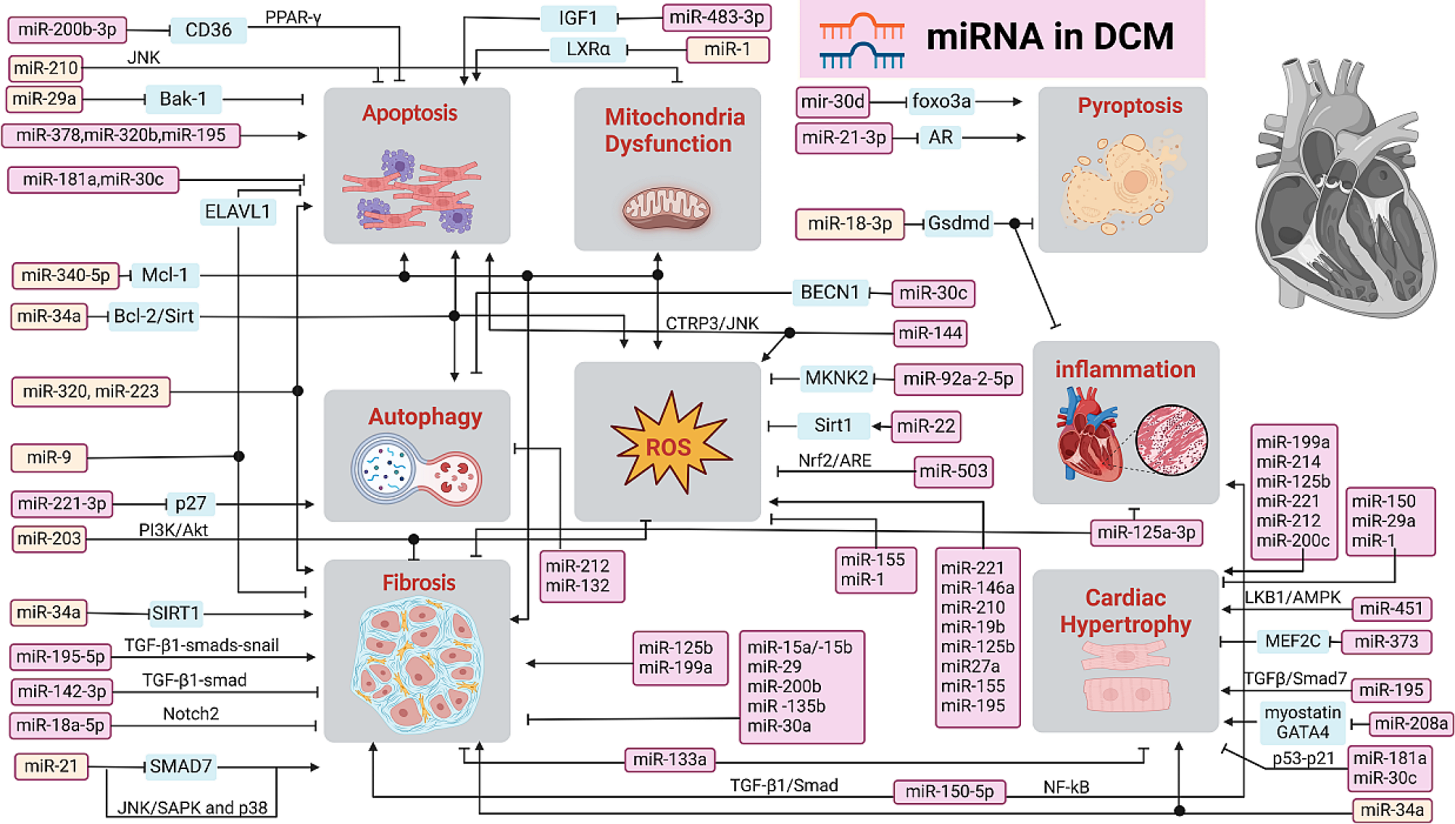
Functional miRNAs promote or inhibit cardiac hypertrophy, fibrosis, ROS, mitochondria dysfunction and cell death in the pathology of diabetic cardiomyopathy

**Fig. 2:**
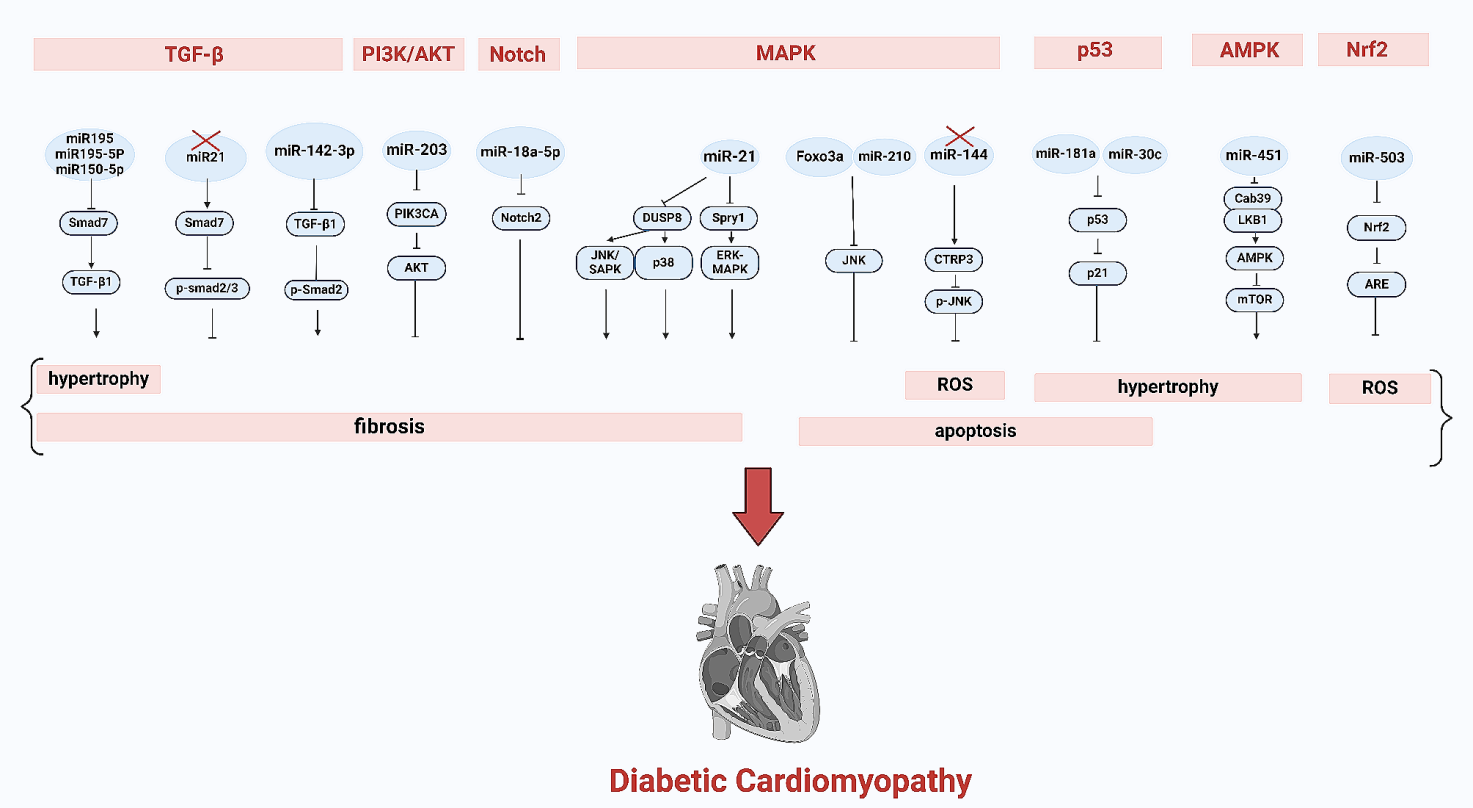
MiRNA regulate fibrosis through TGF-β, PI3K/AKT, Notch, and MAPK signaling pathways, and apoptosis through MAPK and p53. P53, AMPK and TGF-β also mediate the role of miRNAs in cardiac hypertrophy. While Nrf2 signaling pathways is the key in ROS

MicroRNAs (miRNAs) are highly conserved, single-stranded ncRNAs typically consisting of 20–22 nucleotides. The typical functionality of miRNAs is to negatively regulate gene expression by binding to target mRNA, leading to either mRNA degradation or inhibition of translation [20]. MiRNAs play diverse roles encompassing cardiac hypertrophy, cardiomyocyte apoptosis, autophagy and pyroptosis, myocardial fibrosis, oxidative stress, and other pathophysiological processes, Figs. 1 and 2 [21–24]. The expression pattern of miRNAs during DCM were revealed in 2011. Since then, there have been continuous studies on the role of miRNAs in the development and progression of DCM.

### Cardiac hypertrophy

Multiple miRNAs have been identified to modulate cardiac hypertrophy and fibrosis in DCM. Anti-hypertrophic miRNAs, such as miR-1 [25], miR-133a [26], miR-373 [27], miR-181a [28], miR-150 [22], miR-30c [29], miR-378a [30], miR-29a [31] and miR-200c [32]. Pro-hypertrophic miRNAs include miR-208a [33], miR-451 [34], miR-214, miR-212 [35], miR-221 [36], miR-195, miR-125b [37] and miR-199a [38]. For instance, miR-1, a muscle-specific miRNA, attenuates cardiomyocyte hypertrophy by negatively regulating calcium signaling components calmodulin, Gata4 and Mef2a [39]. Overexpression of miR-133a has been shown to prevent hypertrophic changes in DCM by downregulating the serum and glucocorticoid-regulated kinase 1 (SGK1), IGFR1 and myocyte-specific enhancer factor 2C (MEF2C) [26]. Additionally, miR-373 influences MEF2C signaling, a key transcription factor for myocardial hypertrophy and mediates cardiac fibrosis through activation of the p300 gene [27]. MiR-181a and miR-30c could synergistically regulate the p53–p21 pathway in diabetes-induced cardiac hypertrophy [28]. MiR-208a promotes cardiac hypertrophy by inhibiting myostatin, GATA4, and β-myosin heavy chain (MHC) expression [33]. Kuwabara et al. [34] demonstrated that miR-451 could suppress the LKB1/AMPK pathway in cardiac hypertrophy induced by diabetes. Biao *et al.* [40] reported that miR-195 accelerates cardiomyocyte hypertrophy *in vitro* induced by high-glucose through downregulating the expression of Smad7 and modulating TGF-β/Smad pathways. Moreover, the endothelial-to-meschenymal transition (EndMT) is a key driver of cardiac fibrosis and plays an important role in the pathogenesis of DCM. Ding et al. [41] reported that silencing miR-195-5p inhibits the TGF-β1-smads-snail pathway by targeting Smad7, thereby attenuating EndMT and reducing myocardial fibrosis in DCM.

### Myocardial fibrosis

Myocardial fibrosis stands out as a prominent pathological characteristic of DCM, and its regulation involves various miRNAs such as miR-34a, miR-150-5p, miR-18a-5p, miR-30, miR-199 [38], miR-135b [42], miR-133a [23], miR-125b, miR-200b [43], miR-320 [44], miR-15a/b [45], miR-21 [46], miR-29 [47] and so on. For instance, research by Bernardo et al. [48] highlighted the involvement of miR-34a in cardiac fibroblasts exposed to high glucose, showing its ability to enhance collagen synthesis through decreasing the level of sirtuin 1 (SIRT1) [49, 50]. Che et al. [51] indicated that inhibiting miR-150-5p could ameliorate NF-κB-related inflammation and TGF-β1/Smad-induced cardiac fibrosis through targeting Smad7. Li et al. [52] showed that the miR-21 inhibition decreased cardiac perivascular fibrosis by suppressing EndMT and upregulating Smad7 while activating p-Smad2 and p-Smad3. Additionally, inhibition of miR-21 could reduce fibrosis via blocking the activation of the p38 signaling pathway [46, 53]. MiR-18a-5p was found to downregulate Notch2 expression, thereby suppressing EndMT in human aortic valvular endothelial cells exposed to high glucose [54]. Zhu et al. [55] demonstrated that miR-142-3p could attenuate high glucose-induced EndMT in primary human aortic endothelial cells(HAECs), possibly through blocking the TGF-β1/Smad signaling pathway. In addition, Yang et al. [56] showed that the miR-203 may function as a cardioprotective regulator in DCM, as its up-regulation could reduce myocardial hypertrophy, myocardial fibrosis, myocardial apoptosis by targeting PIK3CA via inactivation of PI3K/Akt signaling pathway.

### Mitochondrial damage and oxidative stress

Mitochondrial damage and the accumulation of excessive reactive oxygen species (ROS), including reduced oxygen (O_2_) metabolites, superoxide anion (O_2_^−^), hydroxyl radicals (^·^OH) and hydrogen peroxide (H_2_O_2_), are recognized as significant molecular and cellular mechanisms contributing to cardiac dysfunction and cardiomyopathy in diabetic individuals. Various miRNAs such as miR-340-5p, miR-92a-2-5p, miR-1 [39], miR-22, miR-144, miR-195 [57], miR-200c, miR-221 [36], miR-146a, miR-34a [58], miR-210, miR-19b, miR-125b, miR-155, miR-27a and miR-503 [35] have been implicated in the regulation of hyperglycaemia-induced oxidative stress. For instance, Zhu et al. indicated that overexpression of miR-340-5p [59] in cardiomyocytes led to increased mitochondrial functional loss, oxidative stress, and cardiomyocyte apoptosis in diabetic mice by targeting myeloid cell leukemia 1(Mcl-1). Yu et al. [60] observed that decreased miR-92a-2-5p expression was also detected in high glucose-induced cardiomyocytes. Overexpression of miR-92a-2-5p ameliorated cardiomyocyte oxidative stress injury, by inhibiting MKNK2 expression and leading to decreased phosphorylation of p38-mitogen-activated protein kinase(MAPK) signaling. Overexpression of miR-22 was shown to attenuate oxidative stress by upregulating Sirt 1 in DCM [61]. Furthermore, Yu et al. found that downregulation of miR-144 protected against diabetes-induced cardiac oxidative damage by directly targeting nuclear factor-erythroid 2-related factor 2 (Nrf2) [62]. Members of miR-200 family such as the miR-200a, and miR-200c play a crucial role in oxidative stress in cardiovascular complications of diabetes. MiR-200c was shown to enhance COX-2 expression in endothelial cells by suppressing ZEB1 expression, promoting prostaglandin E2 production, and thereby reducing endothelium-dependent relaxation [63]. Additionally, Miao et al. demonstrated that upregulated expression of miR-503 in DCM was associated with the protective effects of Phase II Enzyme Inducer CPDT via the nuclear factor erythroid 2-related factor 2/anti-oxidant response elements (Nrf2/ARE) signaling pathway, a key anti-oxidant signaling pathway [64, 65].

### Cell death

Apoptosis, autophagy, necrosis and pyroptosis are four pathways resulting in cell death, playing important roles in the pathological progression of DCM. Several miRNAs, including miR-1, miR-30, miR-483-3p, miR-144, miR-21 [66, 67], miR-210, miR-212 [68], miR-200b-3p, miR-195, miR-320b, miR-133, miR-221 [36], miR-320 [69], miR-378 [70], miR-34a [71], miR-29 [47], miR-181a [28], have been associated with cell death. For example, the miR-30 family is one of the most abundant miRNAs in the heart, comprising miR-30a, miR-30b, miR-30c, miR-30d and miR-30e, participates in DCM through a variety of mechanisms, including autophagy, apoptosis, oxidative stress, and inflammation [72, 73]. Enforced expression of miR-30a or miR-30b can inhibit apoptosis induced by hydrogen peroxide, by influencing p53 translation [74]. Conversely, upregulation of miR-30d in DCM has been linked to promoting cardiomyocyte pyroptosis, leading to enhanced proinflammatory cytokines IL-1β and IL-18, as well as caspase-1. In addition, microRNA-30d could regulate cardiomyocyte pyroptosis by directly targeting foxo3a in DCM [24]. Qiao et al. found that miR-483-3p [75] was upregulated in streptozotocin-induced diabetic mice, promoting myocardial cell apoptosis by transcriptionally repressing insulin growth factor 1 (IGF1). Furthermore, repression of miR-144 decreased the protein levels of Bax. It phosphorylated c-Jun amino-terminal kinase (p-JNK) promoted cell proliferation and reduced apoptosis of cardiomyocytes treated with high glucose through targeting the CTRP3/JNK signaling pathway [76]. Moreover, Lin et al. demonstrated that miR-210 [77] repression facilitates advanced glycation end-product (AGE)-induced cardiac mitochondrial dysfunction and apoptosis through JNK activation. On the other hand, low expression of miR-200b-3p in DCM was associated with increased cardiocyte apoptosis, and its overexpression could reduce apoptosis by targeting the CD36/PPAR-γ signaling pathway [78]. Upregulation of miR-195 was reported to lead to cardiomyocyte apoptosis. Zheng et al. revealed that the knockdown of miR-195 could inhibit myocardial hypertrophy in diabetes by preventing cardiomyocyte apoptosis in cardiac endothelial cells in response to non-esterified fatty acid (NEFA) such as palmitate [57]. Additionally, Tang et al. reported that enforced expression of miR-22 could attenuate oxidative injury by upregulating Sirt 1 in diabetic cardiomyopathy [61]. These findings underscore the intricate regulatory roles of miRNAs in modulating cell death pathways and their implications for the pathogenesis of DCM.

## LncRNAs

**Fig. 3:**
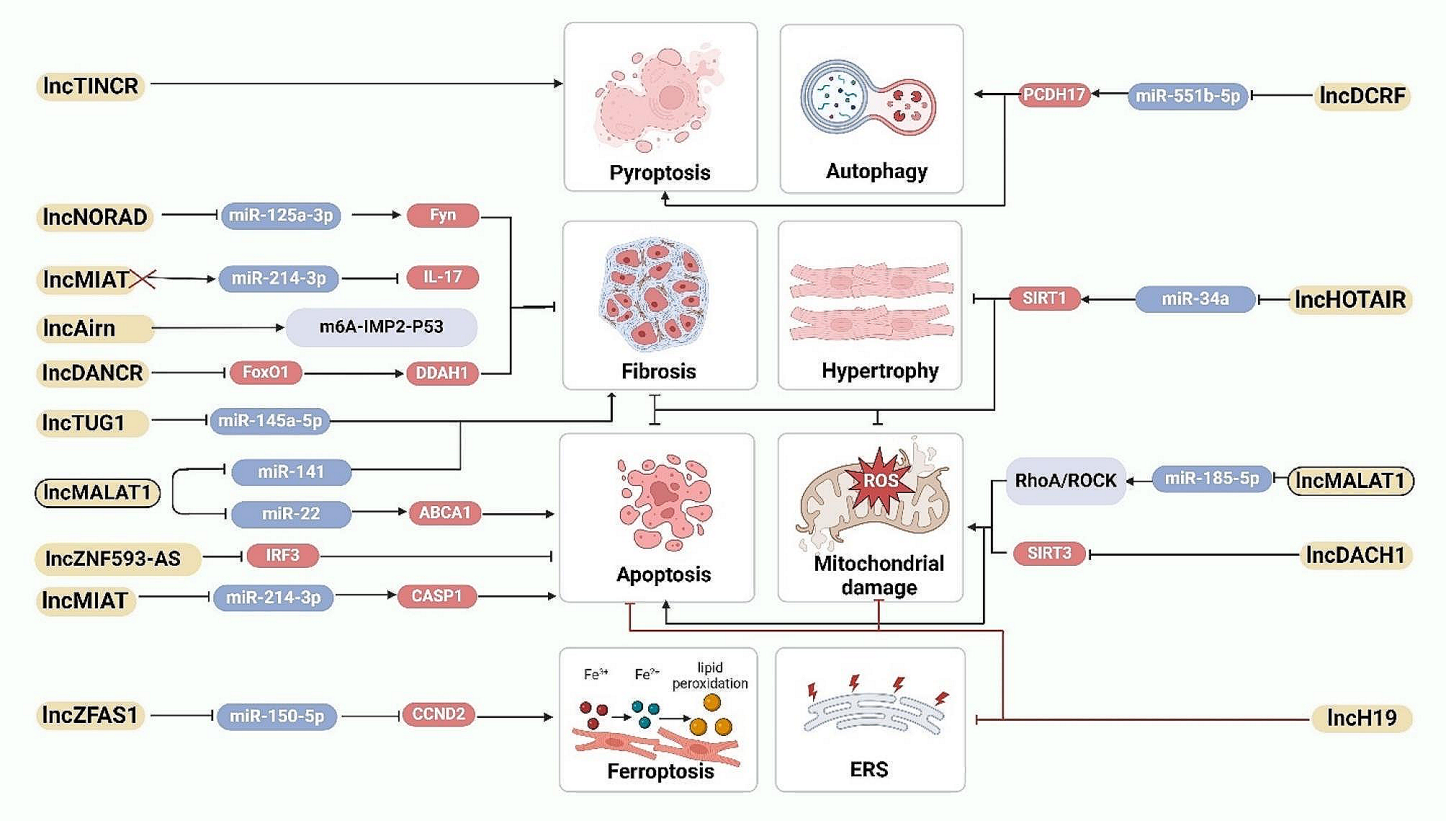
Involvement of lncRNA in the pathogenesis of diabetic cardiomyopathy

lncRNAs were the heterogeneous RNA transcripts, which are longer than 200 nucleotides, and have many epigenetic regulation forms, including DNA methylation, histone modification and regulation of miRNA [79, 80]. lncRNAs play essential roles in multiple biological processes, such as chromatin structural changes, transcriptional regulation, post-transcriptional processing, intracellular trafficking, and regulation of enzyme activity [81, 82]. Recently, growing evidence has suggested that lncRNAs can actively participate in the pathogenesis of diverse cardiovascular diseases, including DCM, Fig. 3.

### Cardiac hypertrophy and myocardial fibrosis

Myocardial fibrosis is a critical pathological change observed in DCM. Feng et al. [83] reported increased lncRNA DCRF expression and induced autophagy in cardiomyocytes in high glucose-induced rats. Knockdown of DCRF was found to reduce cardiomyocyte autophagy, attenuate myocardial fibrosis and improve cardiac function in diabetic rats by targeting miR-551b-5p. In another study, Liu et al. [84] indicated that lncRNA NORAD was upregulated in diabetic and DCM mice. Silencing NORAD expression could reduce inflammatory responses, and improve cardiac function and fibrosis in DCM mice via the ceRNA network of NORAD/miR-125a-3p/Fyn. Moreover, Qi et al. [85] demonstrated that high glucose-induced lncRNA MIAT upregulation was responsible for Interleukin-17 (IL-17) production in cardiomyocytes, which was a pro-inflammatory cytokine and a key regulator of host inflammation. Knockdown of MIAT could significantly attenuate IL-17 expression, ameliorate cardiac fibrosis and improve cardiac contractility. Recent research has also highlighted the involvement of lncRNA Airn in the progression of cardiac fibroblasts in DCM, demonstrating its ability to alleviate diabetic cardiac fibrosis via a m6A-IMP2-p53 axis [86]. EndMT was induced by high glucose and drove to cardiac fibrosis. LncRNA DANCR could markedly attenuate high glucose-mediated EndMT *in vitro* by inhibiting the activation of FoxO1 and increasing the expression of DDAH1 [87]. Moreover, Wang et al. revealed that lncRNA TUG1 was upregulated in diabetic mice exposed to high glucose, TUG1 overexpression promoted myocardial fibrosis by suppressing the expression of microRNA-145a-5p [88]. These studies underscore the intricate regulatory roles of various lncRNAs in modulating myocardial fibrosis and cardiac function in the context of DCM.

### Mitochondrial damage and oxidative stress

Mitochondrial damage and oxidative stress have a significant involvement in the progression of DCM. Recent research has highlighted the upregulation of lncRNA DACH1 in DCM hearts and high glucose-treated cardiomyocytes. DACH1 aggravates DCM by promoting mitochondrial oxidative stress, cell apoptosis, cardiac fibrosis and hypertrophy via increasing ubiquitination-mediated SIRT3 degradation in mice’s hearts [89]. In a study by Gao et al. [50], it was found that lncRNA HOTAIR expression was significantly decreased in diabetic mice hearts. Knockdown of HOTAIR in high glucose-induced H9c2 cells resulted in increased oxidative injury. HOTAIR could protect against DCM via activating of the Sirtuin 1(SIRT1) expression by sponging miR-34a [90]. Additionally, lncRNA MALAT1 was significantly upregulated in the myocardium of diabetic mice and high glucose-induced cardiomyocytes, mediated oxidative stress, mitochondrial damage and apoptosis through activating the RhoA/ROCK pathway via sponging miR-185-5p. LncRNA H19 is a key lncRNA in DCM, which produces a 2.3-kb non-coding mRNA and is conserved via matriarchal evolution [91]. Wang et al. demonstrated that H19 repressed oxidative stress, endoplasmic reticulum stress (ERS) and apoptosis *in vitro*, furthermore, it reduced cardiomyocytes apoptosis and improved fibrosis in vivo through PI3K/AKT/mTOR signaling pathway [92].

### Cell death

Some lncRNAs have been identified to be correlated with cardiomyocyte apoptosis, pyroptosis, ferroptosis and autophagy during the process of DCM. For instance, lncRNAs MALAT1 not only has been implicated in mitochondrial injury, but also participated in cardiomyocyte apoptosis. Zhang et al. [93] reported that Down-regulation of lncRNA MALAT1 could reduce cardiomyocyte apoptosis and improve left ventricular function in diabetic rats. Furthermore, Wang Chong and colleagues [94] found that MALAT1 recruited the histone methyltransferase EZH2 to the promoter region of miR-22, thereby inhibing its expression. EZH2, in turn, upregulated the expression of ATP-binding cassette transporter A1 (ABCA1), a known target gene of miR-22. Knockdown of EZH2 was found to enhance cardiac function and prevent cardiomyocyte apoptosis in db/db mice and mouse cardiomyocytes cultured inhigh glucose conditions in the presence of MALAT1. MALAT1 was involved in the processes of cardiac function and cardiomyocyte apoptosis via the EZH2/miR-22/ABCA1 signaling cascade. lncRNA TINCR participated in pyroptosis in DCM progression, which positively regulated NLRP3 by increasing its mRNA stability, downregulating TINCR could suppress pyroptosis and DCM [95]. Recently, Xie et al. indicated that lncRNA ZNF593-AS directly interacted with the functional domain of interferon regulatory factor 3 (IRF3), thereby inhibiting the fatty acid-induced phosphorylation and activation of IRF3. This interaction ultimately let to mitigation of cardiac cell death and inflammation in DCM [96]. Moreover, lncRNA MIAT was demonstrated to be involve in the progression of cell death in DCM. Xiao et al. [97] reported that MIAT played a vital role in regulating of pyroptosis in DCM via targeting miR-214-3p. Zhou et al. [98] indicated that MIAT knockdown could reduce DAPK2 expression by increasing miR-22-3p, and inhibit apoptosis in cardiomyocytes exposed to high glucose. Ferroptosis is an iron-dependent regulated necrosis associated with a new form of regulatory cell death [99]. Ni et al. [100] showed that inhibition of lncRNA ZFAS1 could alleviate the development of DCM by reducing ferroptosis via stabilizing miR-150-5p to activate CCND2. Interactions of ncRNA are also involved in cardiomyocyte apoptosis. The interaction among NORAD, miRNA-150-5p and ZEB1 has been clarified to influencing the proliferation and apoptosis in HG-induced AC16 cells [101].

## Circular RNAs

**Fig. 4:**
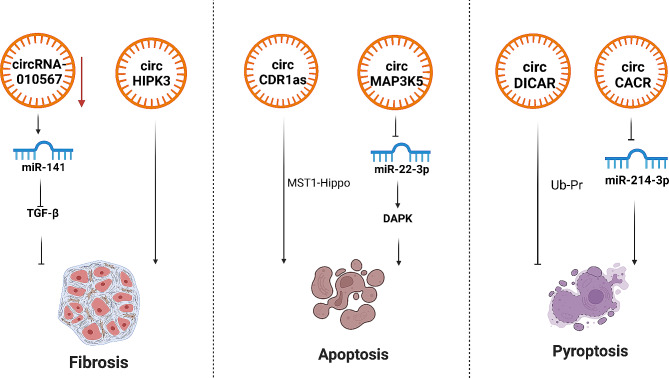
The role of circRNA in the pathogenesis of diabetic cardiomyopathy

CircRNAs are produced from precursor mRNAs by the back-splicing of exons in eukaryotes and are widely expressed in a tissue-specific and developmental stage-specific pattern. However, knowledge of these species has remained limited due to their difficult study through traditional methods of RNA analysis [21, 102]. CircRNAs differ from linear RNAs in that they are circular molecules with covalently closed loop structures, which are involved in a wide range of biological processes, the expression disorder of circRNAs might lead to abnormal cellular functions and disease. CircRNAs may inhibit the translation of mRNAs, altering gene expression by regulating splicing or transcription and by interacting with RNA-binding proteins [103]. However, the regulation of circRNAs in cardiovascular diseases remains largely unexplored Fig. 4.

Compared with miRNAs and lncRNAs, the understanding of circRNAs in the molecular mechanisms of DCM is still needs to be improved. Yuan et al. revealed that circRNA DICAR was downregulated in diabetic mice hearts and was associated with cardiac dysfunction, cardiac cell hypertrophy, and cardiac fibrosis [104]. Yang et al. showed the involvement of another circRNA in the regulation of diabetic myocardial fibrosis. They found that circRNA-0076631 was increased both in high glucose-induced cardiomyocytes and in the serum of diabetic patients, modulated miR-214-3p and its target gene caspase-1 and mediated fibrosis-associated protein resection [105]. Zhou et al. reported that circRNA-010567 sponged miR-141 and upregulated target gene TGF-β1, mediated fibrosis-associated protein resection in the diabetic mice myocardial fibrosis model. Silencing the expression of circRNA-010567 could suppress fibrosis-associated protein resection, including Col I, Col III and α-SMA in the regulation of diabetic myocardial fibrosis [106]. CircRNA homeodomain interacting protein kinase 3 (circHIPK3) is a particularly abundant circRNA involved in metabolic dysregulation and tumorigenesis [107–109]. Wang et al. found that circHIPK3 was upregulated in a DCM model of streptozotocin (STZ)-induced diabetic mice [110]. CircHIPK3 increased the expression of fibrosis-associated genes, such as COL1A2, COL3A1 and α-SMA, via sponging miR-29b-3p in AngII-induced mouse myocardium [111]. Knockdown of circHIPK3 could ameliorate myocardial fibrosis and improve cardiac function in vivo, while decreasing the proliferation of CFs treated with Ang II via miR-29b-3p/Col1a1-Col3a1 *in vitro.* circRNA circular cerebellar degeneration-related protein 1 antisense (circCDR1as) is degraded by sponging miR-671 via protein Argonaute 2 [112]. CircCDR1as was upregulated in DCM hearts of STZ-induced diabetic mice, which promoted cardiomyocyte apoptosis through activating the MST1-Hippo pathway in vivo and in HG-treated primary cardiomyocytes. Knocking down CDR1as inhibited cardiomyocyte apoptosis in DCM [113]. Recently, a novel circRNA mitogen-activated protein kinase kinase kinase 5 (circMAP3K5) was found to regulate apoptosis of cardiomyocytes in DCM. Shen et al. indicated that circMAP3K5 upregulated in in high glucose-induced H9c2 cardiomyocytes, accelerated cardiomyocytes apoptosis through the miR-22-3p/death-associated protein kinase 2 (DAPK2) axis [114]. Fu et al. found that circ-0071269 was significantly overexpressed in H9c2 cells upon treatment with high glucose. Circ_0071269 could promote the development of DCM through the miR-145/GSDMA axis. Knockdown of circ_0071269 promoted cell viability and inhibited the inflammatory response, cytotoxicity, and pyroptosis of H9c2 cells *in vitro* [115].

## Clinical application

NcRNAs are involved in the development and progression of DCM, presenting in the blood are extremely stable and can be potentially used as diagnostic and prognostic biomarkers for cardiovascular diseases, consequently allowing early intervention. Furthermore, ncRNAs are modulating various biological pathways, suggesting that these molecules may be harnessed as a novel therapeutic strategy in treating DCM.

Vildagliptin is an oral hypoglycemic drug that reduces hyperglycemia in T2DM. Li et al. reported that vildagliptin could enhance cardiac function in type 2 diabetic mice by restoring autophagy and alleviated fibrosis through the miR-21/SPRY1/ERK/mTOR pathway [66]. Melatonin is a hormone produced by the pineal gland, and it has extensive beneficial effects on various tissues and organs. Che et al. showed that melatonin administration significantly ameliorated cardiac dysfunction and reduced collagen production via inhibiting lncRNA MALAT1/miR-141-mediated NLRP3 inflammasome and TGF-β1/Smads signaling pathway, while the expression of TGF-β1, p-Smad2, p-Smad3, NLRP3, ASC, cleaved caspase-1, mature IL-1β, and IL-18 were downregulated in the heart of mice with diabetes mellitus following melatonin treatment [116]. Furthermore, melatonin was reported to alleviate cardiac dysfunction and cardiomyocyte apoptosis in diabetic rats, notably by downregulating lncRNA H19/MAPK and upregulating miR-29c levels [117]. Diallyl trisulfide (DATS) is an anti-oxidant in garlic oil, can inhibit stress-induced cardiac apoptosis and can be used as a cardioprotective agent. Lin et al. found that the DATS could mediate AGE-induced cardiac cell apoptosis attenuation by promoting FoxO3a nuclear transactivation to enhance miR-210 expression and regulate JNK activation [77]. Pomegranate peel extract (PPE) exhibits a cardioprotective effect due to its anti-oxidant and anti-inflammatory properties, which could significantly ameliorate cardiac hypertrophy in diabetic rats and increase the survival rate. The protective effect of PPE on DCM could be due to the inhibition of the NLRP3/caspase-1/IL-1β signaling pathway and downregulation of lncRNA-MALAT1 [118]. Berberine (BBR) is a natural compound extracted from a Chinese herb (*Rhizoma coptidis*; known as ‘Huang Lian’ in Chinese). It has been traditionally used in Chinese medicine for treating inflammatory disorders and cardiovascular injury induced by diabetes mellitus [119]. Yang et al. indicated that BBR alleviated DCM by inhibiting miR‑18a-3p-mediated gasdermin D (Gsdmd) activation [120]. Citronellal (CT), a monoterpenoid natural product extracted from the grass plant Citronella, has demonstrated anti-thrombotic, anti-hypertensive and anti-diabetic cardiomyopathy properties. Qiu et al. reported that CT significantly reduced vascular plate area and decreased endothelial lipid and cholesterol deposition in the common carotid artery of mice. CT upregulated the expression of activated protein 2α (AP-2α/TFAP2A) and circRNA_102979 in vascular endothelium. This led to an enhanced binding capability of circRNA_102979 to miR-133a, counteracting the inhibitory effect of miR-133a on target genes. Consequently, this mechanism helped alleviate vascular endothelial injury [121]. Ranolazine, a piperazine derivative approved by the US Food and Drug Administration in 2006 for the treatment of stable angina pectoris, has shown effectiveness in treating cardiovascular disease [122]. Ranolazine increased miR-135b expression in cardiac fibroblasts exposed to high glucose. Furthermore, miR-135b directly interacted with caspase-1. Thereby, ranolazine could reduce pyroptosis, inhibit collagen deposition and improve cardiac function in rats by upregulating miR-135b [42]. Activation of cardiac miR-132 leads to adverse remodeling and pathological hypertrophy. CDR132L, a synthetic antisense oligonucleotide that selectively blocks pathologically elevated miR-132, has shown promisingeffects on heart failure (HF) in the early stage following myocardial infarction (MI) in phase I/II trials [123–125]. There are many opportunities for further advancement in cardiovascular medicine, particularly in the new therapeutics to target ncRNAs for diabetic DCM, through conducting large-animal studies and phase I/II trials involving humans.

## Conclusions

In the present review, we provide an overview of the recent advancements in understanding the role of ncRNAs in the pathogenesis of DCM. Various ncRNAs play crucial roles in regulating cardiomyocyte hypertrophy, myocardial fibrosis, apoptosis and autophagy, oxidative stress and inflammatory response, all of which are key mechanisms associated with DCM, Table 1. With the growing epidemic of diabetes mellitus and its related cardiac complications, the potential of ncRNA as promising attractive biomarkers and therapeutic targets for DCM and heart failure has captured significant attention within the scientific community. The identification and characterizations of ncRNAs and the pathways they influence may pave the way for the development of innovative treatments to manage or combat diabetic cardiomyopathy in the near future.

**Table 1:** Regulation information of ncRNAs in diabetic cardiomyopathy

ncRNAs	Pathological mechanism
miR-92a-2-5p	ROS
miR-9	Apoptosis, fibrosis
miR-503	ROS
miR-483-3p	Apoptosis
miR-451	Cardiac hypertrophy
miR-378	Apoptosis
miR-373	Cardiac hypertrophy
miR-34a	Apoptosis, autophagy, fibrosis, ROS, cardiac hypertrophy
miR-340-5p	Apoptosis, mitochondria dysfunction, fibrosis, ROS
miR-320b	Apoptosis, fibrosis
miR-30d	Pyroptosis
miR-30c	Autophagy, apoptosis, cardiac hypertrophy
miR-30a	Fibrosis
miR-29a	Cardiac hypertrophy, apoptosis
miR-29	Fibrosis
miR-223	Apoptosis, fibrosis
miR-221-3p	Autophagy
miR-221	Cardiac hypertrophy
miR-22	ROS
miR-214	Cardiac hypertrophy
miR-21-3p	Pyroptosis
miR-212	Cardiac hypertrophy, autophagy
miR-210	Apoptosis, mitochondria dysfunction
miR-21	Fibrosis
miR-208a	Cardiac hypertrophy
miR-203	Fibrosis, ROS
miR-200c	Cardiac hypertrophy
miR-200b-3p	Apoptosis
miR-200b	Fibrosis
miR-199a	Cardiac hypertrophy, fibrosis
miR-195-5p	Cardiac hypertrophy, fibrosis
miR-195	Apoptosis, fibrosis, cardiac hypertrophy
miR-18a-5p	Fibrosis
miR-18-3p	Pyroptosis, inflammation
miR-181a	Apoptosis, cardiac hypertrophy
miR-15a/-15b	Fibrosis
miR-155	ROS
miR-150-5p	Inflammation, fibrosis, hypertrophy
miR-150	Cardiac hypertrophy
miR-144	ROS, apoptosis
miR-142-3p	Fibrosis
miR-135b	Fibrosis
miR-133a	Cardiac hypertrophy, fibrosis
miR-132	Autophagy
miR-125b	Cardiac hypertrophy, fibrosis
miR-125a-3p	Inflammation, fibrosis
miR-1	Cardiac hypertrophy, ROS, apoptosis
lncZNF593-AS	Apoptosis
lncZFAS1	Ferroptosis
lncTUG1	Fibrosis
lncTINCR	Pyroptosis
lncNORAD	Fibrosis
lncMIAT	Fibrosis, apoptosis
lncMALAT1	Fibrosis, apoptosis, mitochondria dysfunction
lncHOTAIR	Hypertrophy, fibrosis, apoptosis, mitochondria dysfunction
lncH19	ERS, apoptosis, mitochondria dysfunction
lncDCRF	Pyroptosis, autophagy
lncDANCR	Fibrosis
lncDACH1	Apoptosis, mitochondria dysfunction
lncAirn	Fibrosis

## Data Availability

No datasets were generated or analysed during the current study.

## Abbreviations

ABCA1: ATP binding cassette subfamily A member 1
^·^OH: Hydroxyl radicals
ABCA1: ATP-binding cassette transporter A1
AGE: Advanced glycation end-product
Akt: Akt kinase
AMPK: AMP-activated protein kinase
AP-2α: Activated protein 2α
ARE: Anti-oxidant response elements
ASC: Apoptosis-associated speck-like protein containing a caspase recruitment domain
Bax: BCL2 associated X, apoptosis regulator
BBR: Berberine
Bcl-2: BCL2 apoptosis regulator
Cab39: Calcium binding protein 39
CASP1: Caspase 1
CCND2: Cyclin D2
CD36: CD36 molecule
circCDR1as: CircRNA circular cerebellar degeneration-related protein 1 antisense (circCDR1as)
circHIPK3: CircRNA homeodomain-interacting protein kinase 3
circMAP3K5: CircRNA mitogen-activated protein kinase kinase kinase 5
circRNA: Circular RNA
Col I: Collagen I
Col III: Collagen III
Col1a2: Collagen Type I Alpha 2 Chain
Col3a1: Collagen Type III Alpha 1 Chain
COX-2: Cyclooxygenase-2
CPDT: Phase II Enzyme Inducer
CT: Citronellal
CTRP3: C1QTNF3 C1q and TNF related 3
DAPK2: Death-associated protein kinase 2
DATS: Diallyl trisulfide
DCM: Diabetic cardiomyopathy
DDCH1: Dimethylarginine dimethylaminohydrolase 1
DUSP8: Dual specificity phosphatase 8
ELAVL1: ELAV like RNA binding protein 1
EndMT: Endothelial-to-meschenymal transition
ERS: Endoplasmic reticulum stress
EZH2: Enhancer of zeste 2 polycomb repressive complex 2 subunit
FoxO1: Forkhead box O1
foxo3a: Forkhead box O3
Fyn: Fyn proto-oncogene
Gata4: GATA binding protein 4
GSDMA: Gasdermin A
Gsdmd: Gasdermin D
H2O2: Hydrogen peroxide
HAECs: Human aortic endothelial cells
IGF1: Insulin like growth factor 1
IL-17: Interleukin-17
IL-18: Interleukin-18
IL-1β: Interleukin-1β
IMP2: Insulin-like growth factor 2 mRNA binding protein 2
IGF1: Insulin growth factor 1
IRF3: Interferon regulatory factor 3
LKB1: Liver kinase B1
lncRNA: Long ncRNA
lncRNA DACH1: LncRNA dachshund homolog 1
lncRNA DANCR: LncRNA Differentiation antagonizing nonprotein coding RNA
lncRNA DCRF: LncRNA DCM-related factor
lncRNA HOTAIR: LncRNA HOX transcript antisense RNA
lncRNA MIAT: LncRNA myocardial infarction associated transcript
lncRNA NORAD: LncRNA non-coding RNA activated by DNA damage
lncRNA TINCR: LncRNA terminal differentiation-induced non-coding RNA
lncRNA TUG1: LncRNA taurine-upregulated gene 1
lncRNA ZFAS1: LncRNA zinc finger NFX1-type containing 1 antisense RNA 1
lncRNA ZNF593-AS: LncRNA Zinc Finger Protein 593 AS
LncRNAs MALAT1: LncRNA metastasis associated lung adenocarcinoma transcript 1
LXRα: Liver X receptor α
m6A: N6-methyladenosine
MAPK: Mitogen-activated protein kinase
Mcl-1: Myeloid cell leukemia 1
Mef2a: Myocyte enhancer factor 2A
MEF2C: Myocyte-specific enhancer factor 2C
MHC: Myosin heavy chain
miRNA: Micro RNA
MKNK2: MAPK interacting serine/threonine kinase 2
MST1: Macrophage stimulating 1
mTOR: Mammalian target of rapamycin
ncRNAs: Non-coding RNAs
NEFA: Non-esterified fatty acid
NLRP3: NLR family pyrin domain containing 3
Notch2: Notch receptor 2
Nrf2: Nuclear factor-erythroid 2-related factor 2
O_2_: Oxygen
O_2_^−^: Superoxide anion
p21: Cyclin-dependent kinase inhibitor p21
p27: P27 protein
p38: Mitogen-activated protein kinase 14
p53: Tumor suppressor gene p53
PCDH17: Protocadherin 17
PI3K: Phosphatidylinositol 3-kinase
PIK3CA: Phosphatidylinositol-4,5-bisphosphate 3-kinase catalytic subunit alpha
p-JNK: Phosphorylated c-Jun amino-terminal kinase
PPAR-γ: Peroxisome proliferator activated receptor gamma
PPE: Pomegranate peel extract
p-Smad2: Phosphorylated SMAD family member 2
p-Smad3: Phosphorylated SMAD family member 3
RhoA: Ras homolog family member A
ROCK: Rho associated coiled-coil containing protein kinase
ROS: Reactive oxygen species
SAPK: Mitogen-activated protein kinase 9
SGK1: Serum and glucocorticoid-regulated kinase 1
SIRT1: Sirtuin 1
SIRT3: Sirtuin 3
Smad7: SMAD family member 7
SPRY1: Sprouty RTK signaling antagonist 1
STZ: Streptozotocin
T2DM: Type 2 diabetes mellitus
TFAP2A: Transcription factor AP-2 alpha
TGF-β: Transforming growth factor beta
ZEB1: Zinc finger E-box binding homeobox 1
α-SMA: α-Smooth muscle actin
